# The Acute Effect of Diesel Exhaust Particles and Different Fractions Exposure on Blood Coagulation Function in Mice

**DOI:** 10.3390/ijerph18084136

**Published:** 2021-04-14

**Authors:** Jian Lei, Zhouzhou Li, Xingke Huang, Xin Li, Guangzheng Zhang, Haidong Kan, Renjie Chen, Yuhao Zhang

**Affiliations:** 1 Key Lab of Public Health Safety of the Ministry of Education and NHC Key Laboratory of Health Technology Assessment, School of Public Health, Fudan University, Shanghai 200032, China; 20111020034@fudan.edu.cn (J.L.); 18211020069@fudan.edu.cn (Z.L.); Huangxingke21@163.com (X.H.); kanh@fudan.edu.cn (H.K.); chenrenjie@fudan.edu.cn (R.C.); 2 Department of Neurology, Zhongshan Hospital, Fudan University, Shanghai 200032, China; li.xin2@zs-hospital.sh.cn (X.L.); zhang.guangzheng@zs-hospital.sh.cn (G.Z.); 3 National Clinical Research Center for Interventional Medicine, Shanghai 200032, China

**Keywords:** diesel exhaust particles, air pollution, blood coagulation, bleeding time, prothrombin

## Abstract

The toxicity and widespread exposure opportunity of diesel exhaust particles (DEP) has aroused public health concerns. This study aimed to investigate the acute effect of DEP and different fractions exposure on blood coagulation function in mice. In this study, nine- week-old C57BL/6J male mice were divided into four exposure groups (with 15 mice in each group). The water-soluble (WS) and water-insoluble (WIS) fractions of DEP were isolated, and intratracheal instillation was used for DEP, WS and WIS exposure. The phosphate buffer saline (PBS) exposure group was set as the control group. After 24 h exposure, the mice were sacrificed for blood routine, coagulation function and bleeding time examinations to estimate the acute effect of DEP, WS and WIS exposure on the blood coagulation function. In our results, no statistically significant difference in weight of body, brain and lung was observed in different exposure groups. While several core indexes in blood coagulation like bleeding time (BT), fibrinogen (FIB), activated partial thromboplastin time (APTT) and prothrombin time (PT) altered or showed a lower tendency after DEP, WS and WIS exposure. For example, BT was lower In WIS exposure group (211.00 s) compared with PBS exposure group (238.50 s) (*p* < 0.01), and FIB was lower in WS exposure group (233.00 g/L) compared with PBS exposure group (249.50 g/L) (*p* < 0.05). Additionally, systemic inflammation-related indexes like white blood cell count (WBC), neutrophil count (NEUT), lymphocyte count (LYMPH) altered after DEP, WS and WIS exposure. In conclusion, DEP, WS and WIS fractions exposure could result in the hypercoagulable state of blood in mice. The noteworthy effects of WS and WIS fractions exposure on blood coagulation function deserve further investigation of the potential mechanism.

## 1. Introduction

Air pollution is a significant public health concern globally, and it can result in reduced health levels of residents throughout the lifetime [1,2]. Based on the Global Burden of Disease Study in 2015, cardiovascular disease (CVD) hospitalization and mortality attributable to fine PM air pollution continues to be one of the greatest public health concerns worldwide [3,4]. Moreover, the associations between adverse health effects and ambient air pollution have been identified [5,6]. Exposure to air pollution is known to impair cardiovascular function, exacerbate disease and increase CVD mortality [7,8]. The toxicity and widespread exposure opportunity of air pollution arouse public health concerns about its damage effects. In urban areas, motor vehicle emissions and the power industry were the main sources of both primary pollutants and secondary pollutants [9,10]. Diesel exhaust particles (DEP) are rich in combustion-derived nanoparticles that make up a significant proportion of urban fine and ultrafine particulate matter [11].

A previous study discovered that DEP exposure was related to several pathological changes related to CVD [12]. DEP exposure was able to increase central arterial stiffness and diastolic blood pressure [13]. Hypercoagulable blood could be one of the important risk factors that contributes to the increased incidence and mortality of CVD [8,14]. The direct and primary exposure route of DEP is inhalation by the respiratory system, correspondingly, that DEP exposure could increase the susceptibility of the lung to infection by depressing the antimicrobial potential of alveolar macrophages [15,16]. DEP instilled through the airway was able to induce adverse effects such as inflammation, oxidative damage and coagulopathy [17]. In the mouse model, thrombosis and prothrombotic effects were enhanced in DEP inhalation exposure [18,19]. Research in humans also discovered that ex vivo thrombus formation increased in short-term DEP exposure [20]. However, several other studies have failed to show any association between blood coagulation function and DEP exposure [21,22], and research on the acute effect of DEP exposure is relatively limited. 

In this study, we aimed to estimate the effects of DEP, water-soluble (WS) and water-insoluble (WIS) fractions of DEP exposure on blood coagulation in the mouse model. Once the relationship between DEP exposure and blood coagulation state is confirmed at experimental levels, it is possible to raise awareness and reduce the mortality risk of CVD in advance. 

## 2. Materials and Methods

### 2.1. Exposure Sample Preparation

**Figure 1:**
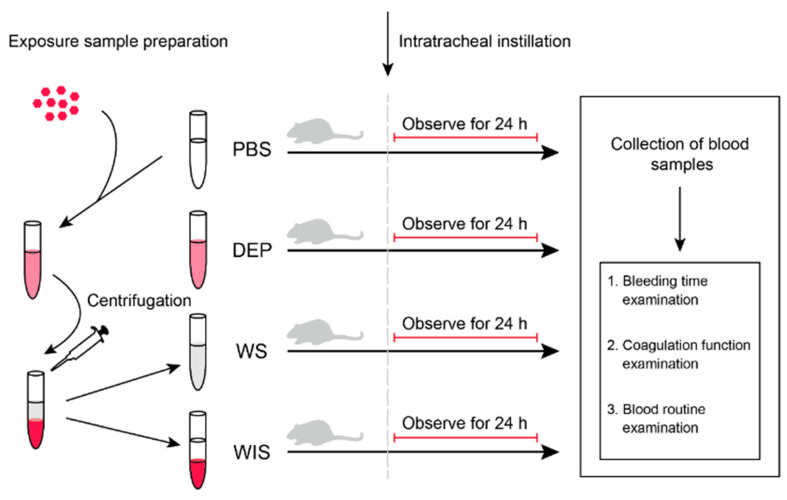
The procedure of exposed samples preparation and the experiment. Phosphate buffer saline (PBS) exposure group, DEP: diesel exhaust particles (DEP) exposure group, water-soluble (WS) fractions of DEP exposure group, and water-insoluble (WIS) fractions of DEP exposure group.

The procedure of exposed sample preparation is shown in Figure 1. DEP standard substance (SRM2975, NIST, USA) was obtained from the National Institute of Standards and Technology (NIST), and added in PBS buffer (10010023, Thermo Fisher Scientific, USA) at the concentration of 2 mg/mL for exposure. To obtain the WS fraction of DEP, the DEP solution was well mixed with vortex for 30 s, and centrifuged for 30 min at 12,000× *g*. Then the supernatant was transferred to a new tube as WS fraction of DEP. The residuals were resuspended with PBS as WIS fraction of DEP. The DEP, WS and WIS exposed samples were diluted in the same volume. All the exposed samples were stored at the temperature of −80 °C and kept away from direct sunlight before intratracheal administration. 

### 2.2. Animals and Study Design

In this study, 9week-old C57BL/6J male mice (body weight 26.86 ± 1.45 g, SPF class) were obtained from the Department of Laboratory Animal Science, Shanghai Medical College of Fudan University (Shanghai, China). Before the experiment, the mice were randomly divided into 4 exposure groups (PBS, DEP, WS and WIS exposure groups) with 15 mice in each group. Mice were allowed free access to standard diets [Mouse maintenance food (reference A03.10, SAFE)] and sterilized water [23]. The mice were fed in controlled environment conditions (12 h light-dark cycle, temperature 20–25 °C, humidity 40–70%, SPF). After adaptive feeding for a week, the mice were exposed to PBS, DEP, WS and WIS by intratracheal administration. All the procedures in this study were approved by the Animal Care and Use Committee of Fudan University (no. 201802176S), and all the mice were treated under the principles of laboratory animal care (NIH publication no. 85–23, revised in 1985). 

### 2.3. Intratracheal Instillation

Intratracheal instillation was used for DEP exposure as it could simulate the real exposure of the respiratory system, and additionally the actual dose delivered to the lungs of each animal could be accurate. To ensure the exposed samples were all in homogeneous distribution, all the samples were treated with ultrasonic vibrator (Sonic Ultrasonic Instrument, China) for 30 min and then mixed with vortex for 30 s before the intratracheal instillation. For DEP, WS and WIS intratracheal instillation, the mice were treated with isoflurane (3%) for inhalation anesthesia and placed supine with extended neck on an angled operating board. A cannula (18 Gauge, Becton Dickinson, USA) was inserted via the mouth into the trachea. The exposed samples (PBS, DEP, WS, WIS exposure groups with 20 µL each) were intratracheally instilled via a sterile syringe and followed by 150 μL air bolus. The exposed dose was based on previous animal experiments for DEP intratracheal instillation [19,24]. Finally, the intubation catheter was removed and the mouse transferred to a vertical hanging position with the head up for 5 min, to ensure that the delivered exposure was maintained in the lung and did not block the airways [25]. As we aimed to investigate the acute effect of DEP exposure on blood coagulation, we sacrificed the mice for analyses after 24 h observation. The 24 h exposure time was also used in the previous investigation on the acute effect of air pollution [26,27,28].

### 2.4. Collection of Blood Samples and Organs

Before blood sample collection, the body weight of each mouse was weighed and recorded with electronic balance (Shanghai Sunny Hengping Scientific Instrument, China). Full blood samples of mice were collected by extirpating eyeballs. Automatic blood coagulation analyzer (C2000, Perlong Medical, China) was used for the blood coagulation examination. Automatic hematology analyzer (Sysmex, Kobe, Japan) was used for blood routine examination. After sacrifice, the organs of mice were collected and weighed with electronic balance. The organs were flash-frozen with liquid nitrogen and stored at a temperature of −80 °C. 

### 2.5. Blood Routine, Coagulation Function and Bleeding Time Examinations

The blood routine examination included red blood cell count (RBC), platelet (PLT), white blood cell count (WBC), neutrophil count (NEUT), lymphocyte count (LYMPH), monocyte count (MONO), eosinophil count (EO), basophil count (BASO), hemoglobin (HGB), mean corpuscular hemoglobin (MCH) and mean corpuscular hemoglobin concentration (MCHC). The coagulation function examination included prothrombin time (PT), thrombin time (TT), activated partial thromboplastin time (APTT) and fibrinogen (FIB). The mice were fixed in the operating fixator for the examination of bleeding time (BT). After disinfection with 75% alcohol, 0.2–0.3 cm tail of each mouse was cut off and the duration from the transection moment to bleeding stopped was measured and recorded. 

### 2.6. Statistical Analysis

Statistical analyses were conducted using R software (Version 4.0.2, R Foundation for Statistical Computing, Vienna, Austria). Kruskal-Wallis test was used to compare the statistically significant difference, and the results were expressed as median, min and max. *p*-value less than 0.05 was regarded as statistical significance. 

## 3. Results

### 3.1. The Results of Body Weight and Organ Weight

As displayed in Table 1, no statistically significant difference in body weights of mice was observed between DEP and PBS exposure groups, WS and PBS exposure groups and WIS and PBS exposure groups. At the same time, we also analyzed the weights of lung and brain. Similar to the bodyweight results, no statistically significant difference in lung or brain weights was observed between DEP and PBS exposure groups, WS and PBS exposure groups and WIS and PBS exposure groups. These results indicated that the DEP, WS or WIS exposure did not show an acute effect on the body weight, brain and lung weights of mice. 

### 3.2. The Results of Bleeding Time and Coagulation Function Examinations

As displayed in Table 2, the BT in the DEP exposure group was lower compared with PBS exposure group, but the difference was not in statistical significance. A similar lower tendency was also observed in coagulation function indexed like APTT and PT. The BT in WS exposure group was lower compared with PBS exposure group (*p* < 0.05). At the same time, FIB was lower in WS exposure group compared with PBS exposure group (*p* < 0.05). A similar lower tendency was also observed in PT and APTT. In WIS exposure group, BT was lower compared with PBS exposure group, and the difference was in statistical significance (*p* < 0.01). A similar lower tendency was also observed in coagulation function indexed like APTT, FIB, PT and TT. The results indicated that the acute effect of DEP, WS and WIS exposure showed a negative influence on BT. Additionally, the core coagulation function indexed like FIB and TT also altered after DEP, WS and WIS exposure. 

### 3.3. The Results of Blood Routine Examination

The result of the blood routine examination is demonstrated in Table 3. In DEP exposure groups, PLT number was higher compared with PBS exposure groups, but no statistically significant difference was observed. Other key indexes like WBC and LYMPH also were higher in DEP exposure group. In WS exposure group, PLT number was higher compared with PBS exposure group, but no statistically significant difference was observed. Indexes like WBC, NEUT and LYMPH were relatively higher in WS exposure group. In WIS exposure group, PLT number was lower compared with PBS exposure group (*p* < 0.05). Other key indexes like RBC, NEUT, WBC and LYMPH were relatively higher in WIS exposure group. These results indicated that WIS of DEP exposure showed a negative influence on PLT number, and indexed related to inflammation response like WBC, NEUT and LYMPH also altered after DEP, WS and WIS exposure.

## 4. Discussion

In this study, we isolated the WS and WIS fractions of DEP to investigate the acute effect on blood coagulation of DEP, WS and WIS exposure. Intratracheal instillation was used to simulate the real exposure route of DEP and different fractions. Blood routine, coagulation function and bleeding time examinations were used to quantitatively estimate the blood coagulation function and systemic inflammation levels. Based on our results, the DEP, WS and WIS exposure did not show an acute effect on the body or organ weight of the mice. At the same time, the core indexes of blood coagulation altered or showed consistent tendency after DEP, WS and WIS exposure, which provided evidence for the acute effect of DEP, WS and WIS fractions exposure on blood coagulation in mice. 

BT is a coagulation-related index that indicates the comprehensive endogenous and exogenous coagulation system function. Shortened BT was able to reflect the hypercoagulability state of the blood. In our results, BT was shortened after WS and WIS exposure, and BT after DEP exposure also showed a lower tendency. Our findings were consistent with the previous experimental studies that BT was lower after DEP exposure in the rat model [29], Similar results were also observed in the mouse model after DEP exposure [30]. Thrombosis is the most common underlying pathology triggering CVD and stroke [8]. Additionally, increased formation of ex vivo thrombus and decreased thrombotic occlusion time were also observed in the human and mouse after short-term DEP exposure [18,20]. These results indicated WS and WIS fraction exposure showed a significantly adverse effect on BT compared with DEP exposure. Research also discovered that it is the surface constituents of the PM rather than the carbon core that appears to be especially detrimental [22]. In our study, the extraction procedure of WS and WIS fraction from DEP could expose the surface constituents of DEP and caused a significant adverse effect on blood coagulation function. 

APTT can reflect the clotting activity of the endogenous coagulation system, and PT is an indicator reflecting the activity of coagulation factors Ⅰ, Ⅱ, Ⅴ, Ⅶ and Ⅹ in peripheral blood. TT is used to diagnose blood coagulation disorders and indicates an abnormality in the conversion of FIB to fibrin. In our results, APTT and PT exerted lower tendency in DEP, WS and WIS exposure groups compared with PBS exposure group. TT showed a lower tendency in WIS group compared with PBS exposure group. These indexed indicated that the mice showed pro-coagulation tendency after DEP, WS and WIS exposure. Consistent with our results, APTT and PT were observed to be shortened in the mouse model after DEP and different fractions exposure [18,19,31,32]. While the tendencies in our results were relatively slight compared with other research. This diversity could be attributed to the different exposure doses and time between studies. But the shorten tendency is consistent with the enhanced coagulation trend of DEP exposure. 

FIB is an essential coagulation glycoprotein that helps in the formation of blood clots. In our results, FIB was lower after WS exposure compared with PBS exposure, and the difference is in statistical significance. Meanwhile, FIB in WIS exposure groups also showed a lower tendency. Some studies observed higher FIB in blood plasma after DEP exposure [19,33]. While several studies failed to find any consistent association or relationship between FIB and air pollution exposure [21,31]. Consistent with our results, several studies discovered a negative association between FIB and PM exposure [34,35]. These results could be caused by excessive consumption of FIB. In blood coagulation procedures, FIB is indispensable for fibrin formation. When the FIB reserved in peripheral blood is not sufficient for fibrin formation to complete blood coagulation, it could decrease in coagulation function examination. 

PLT is cytoplasmic fragments shedding from megakaryocyte, which is of vital importance in the formation of coagulation. Aggregated PLT can block a bleeding vessel and provide a surface for fibrin to form organized clots. In our results, the PLT number was lower in WIS exposure group compared with PBS exposure group, and the difference is in statistical significance. The PLT numbers in DEP and WIS exposure groups showed a higher tendency. Consistent with our results, several studies observed higher PLT number after DEP and air pollution exposure [18,36]. However, research reported that DEP exposure led to a dose-dependent decrease in PLT numbers [30]. The diversity might due to the exposure time and doses varied between studies. Another potential reason could be that the PLT consumption for the aggregated PLT formation is not able to match with the fresh PLT generation, which might result in the reduction of PLT number in blood routine examination. Research also reported that active PLT aggregation occurred in the mouse model after DEP exposure [30,32]. Additionally, it has been shown that the exposure of PM could induce the aggregation of PLT and increase thrombin generation [37]. 

It should be noted that the response of the coagulation system and resultant vascular thrombotic events induced by DEP and the fractions is complicated [38]. Enhanced systemic inflammation by the interaction between DEP and the immune system can also influence the procedure of blood coagulation. It was reported that DEP exposure was related to the up-regulation of allergic inflammation and oxidative stress [39]. Additionally, a positive correlation between C-reactive protein (CRP) and air pollution exposure was observed [36]. In our results, the inflammation-related indexes like WBC, NEUT and LYMPH altered after DEP, WS and WIS exposure. These results are consistent with the hypothesis that inflammation and oxidative stress could be implicated as a mechanism underlying air pollution induced pro-thrombotic and anti-fibrinolytic effects [8]. Further investigations are still needed to ascertain the adverse blood coagulation effects and mechanisms of DEP and different fractions exposure. 

There are several limitations in this study. Firstly, this study investigated the acute effect of DEP and different fractions exposure on blood coagulation, while the long-term effect is also essential to estimate the adverse health effect of DEP and different fractions exposure. Secondly, the animal experiment used in this study was not able to simulate the duration and dose of real human exposure. Thirdly, we did not monitor the consumption of standard diets for mice in this study, which might have a potential influence on the results. Still, this study provides first-hand experimental information for the acute effect on blood coagulation of DEP and different fractions exposure, and the results are the fundamental materials for further mechanism investigation. 

## 5. Conclusions

In conclusion, this study investigated the acute effect on blood coagulation of DEP and different fractions exposure using intratracheal instillation in mice. Based on our results, DEP, WS and WIS fractions exposure could result in the hypercoagulable state of blood in mice. Additionally, up-regulated inflammation response might play an important role in the acute effect on blood coagulation induced by DEP and different fractions exposure. Further studies are still needed to investigate the potential mechanism of DEP, WS and WIS fractions exposure on blood coagulation function. 

## Figures and Tables

**Table 1:** The weights of body and organs in C57BL/6J mice after PBS, DEP, WS and WIS exposure.

Weight After Intervention	PBS			DEP			WS			WIS		
	Median	Min	Max	Median	Min	Max	Median	Min	Max	Median	Min	Max
Body weight	27.14	23.52	30.97	26.92	24.70	27.82	27.60	25.03	29.09	26.95	24.26	28.83
Brain weight	0.46	0.44	0.49	0.45	0.43	0.47	0.46	0.42	0.48	0.45	0.41	0.47
Lung weight	0.38	0.32	1.51	0.39	0.31	1.52	0.38	0.31	1.51	0.42	0.32	1.61

The results are given as median, min and max. Phosphate buffer saline exposure group, diesel exhaust particles exposure group, water-soluble fractions of DEP exposure group. water-insoluble fractions of DEP exposure group, prothrombin time (PT), thromboplastin time (TT), activated partial thromboplastin time (APTT), fibrinogen (FIB) and bleeding time BT).

**Table 2:** The values of blood coagulation parameters in C57BL/6J mice after PBS, DEP, WS and WIS exposure.

Blood Coagulation Parameters	PBS			DEP			WS			WIS		
	Median	Min	Max	Median	Min	Max	Median	Min	Max	Median	Min	Max
PT (s)	7.90	7.10	8.10	7.70	7.20	8.60	7.80	7.10	8.30	7.70	7.10	8.10
TT (s)	14.60	13.00	15.90	14.75	13.40	16.80	14.75	13.50	16.90	14.00	12.90	15.20
APTT (s)	25.45	21.50	27.40	24.45	21.70	28.20	24.10	21.30	27.30	24.50	22.30	27.10
FIB (g/L)	249.50	168.00	692.00	253.00	188.00	332.00	214.50 *	186.00	257.00	233.00	190.00	326.00
BT (s)	238.50	201.00	280.00	230.00	163.00	272.00	218.00 *	161.00	258.00	211.00 **	177.00	236.00

The results are given as median, min and max. Phosphate buffer saline exposure group, diesel exhaust particles exposure group, water-soluble fractions of DEP exposure group, water-insoluble fractions of DEP exposure group, prothrombin time, thromboplastin time, activated partial thromboplastin time, fibrinogen, bleeding time, * *p* < 0.05; ** *p* < 0.01.

**Table 3:** The values of hematological parameters of complete blood analysis in C57BL/6J mice after PBS, DEP, WS and WIS exposure.

Hematology Parameters	PBS			DEP			WS			WIS		
	Median	Min	Max	Median	Min	Max	Median	Min	Max	Median	Min	Max
RBC (×10^12^/L)	8.90	7.85	10.40	8.89	6.01	9.43	8.65	6.32	9.43	8.75	6.47	9.44
PLT (×10^9^/L)	539.00	165.00	877.00	544.00	264.00	733.00	545.50	351.00	692.00	477.00 *	178.00	632.00
WBC (×10^9^/L)	5.18	1.98	7.96	5.84	3.93	8.66	5.72	3.52	9.00	5.31	2.05	8.24
NEUT (×10^9^/L)	0.75	0.30	2.00	0.75	0.40	2.80	0.80	0.60	2.90	0.70	0.30	1.30
LYMPH (×10^9^/L)	4.10	1.40	6.90	4.90	2.50	8.00	4.90	2.20	7.20	4.60	1.70	7.50
MONO (×10^9^/L)	0.02	0.00	0.17	0.02	0.00	0.13	0.02	0.00	0.10	0.02	0.00	0.07
EO (×10^9^/L)	0.00	0.00	0.04	0.00	0.00	0.01	0.00	0.00	0.01	0.00	0.00	0.06
BASO (×10^9^/L)	0.01	0.00	0.03	0.01	0.00	0.03	0.01	0.00	0.03	0.01	0.00	0.01
HGB (g/L)	130.50	111.00	145.00	128.00	84.00	134.00	128.00	91.00	134.00	126.00	90.00	136.00
MCH (pg)	14.20	13.90	15.20	14.35	13.80	15.00	14.20	13.80	15.10	14.15	13.90	15.30
MCHC (g/L)	287.50	277.00	304.00	287.50	273.00	303.00	290.00	280.00	305.00	289.00	284.00	306.00

The results are given as median, min and max. Phosphate buffer saline exposure group, diesel exhaust particles exposure group, water-soluble fractions of DEP exposure group, water-insoluble fractions of DEP exposure group, red blood cell count (RBC), platelet (PLT), white blood cell count (WBC), neutrophil count (NEUT), lymphocyte (LYMPH) count, monocyte (MONO) count, eosinophil (ER) count, basophil (BASO) count, hemoglobin (HGB), mean corpuscular hemoglobin (MCH), mean corpuscular hemoglobin concentration (MCHC). * *p* < 0.05.

## Data Availability

The data used to support the findings of this study are available from the corresponding author upon request.
